# Thin Film Nanocomposite Membranes Based on Zeolitic Imidazolate Framework-8/Halloysite Nanotube Composites

**DOI:** 10.3390/membranes14010007

**Published:** 2023-12-25

**Authors:** Yan Wang, Shaofan Duan, Huixian Wang, Can Wei, Lijuan Qin, Guanying Dong, Yatao Zhang

**Affiliations:** 1 School of Chemical Engineering, Zhengzhou University, Zhengzhou 450001, China; 18608613723@163.com (Y.W.); dsf18339696193@163.com (S.D.); ljqin@ipezz.ac.cn (L.Q.); dongguanying@zzu.edu.cn (G.D.); 2 School of Material Science and Engineering, North China University of Water Resources and Electric Power, Zhengzhou 450046, China; 3 Pollution Prevention and Control Office, Ecological Environment Protection Commission of Zhengzhou, Zhengzhou 450007, China; y.g1145@163.com; 4 Research Department of New Energy Technology, Zhengzhou Institute of Emerging Industrial Technology, Zhengzhou 450046, China

**Keywords:** halloysite nanotubes, ZIF-8, thin film nanocomposite membrane, dye/salt separation

## Abstract

Thin film nanocomposite (TFN) membranes have proven their unrivaled value, as they can combine the advantages of different materials and furnish membranes with improved selectivity and permeability. The development of TFN membranes has been severely limited by the poor dispersion of the nanoparticles and the weak adhesion between the nanoparticles and the polymer matrix. In this study, to address the poor dispersion of nanoparticles in TFN membranes, we proposed a new combination of m-ZIF-8 and m-HNTs, wherein the ZIF-8 and HNTs were modified with poly (sodium p-styrenesulfonate) to enhance their dispersion in water. Furthermore, the hydropathic properties of the membranes can be well controlled by adjusting the content of m-ZIF-8 and m-HNTs. A series of modified m-ZIF-8/m-HNT/PAN membranes were prepared to modulate the dye/salt separation performance of TFN membranes. The experimental results showed that our m-ZIF-8/m-HNT/PAN membranes can elevate the water flux significantly up to 42.6 L m^−2^ h^−1^ MPa^−1^, together with a high rejection of Reactive Red 49 (more than 80%). In particular, the optimized NFM-7.5 membrane that contained 7.5 mg of HNTs and 2.5 mg of ZIF-8 showed a 97.1% rejection of Reactive Red 49 and 21.3% retention of NaCl.

## 1. Introduction

A lack of clean and fresh water is one of the most pervasive problems that afflicts people throughout the world [1,2]. Membrane separation technology has undergone fast development over the past few decades, as it is a promising strategy to tackle the increasing demand for clean water [3,4,5,6]. Recently, significant advances in nanotechnologies and nanomaterials have offered us tremendous opportunities to design and prepare high-performance membranes that can overcome the limitation of the “trade-off” effect between permeability and selectivity [7,8,9]. In particular, thin film nanocomposite (TFN) membranes have attracted tremendous attention given their low resistance, ultrafast water permeation, and precise ionic sieving [10,11,12]. The addition of nanofillers can endow these membranes with improved hydrophilicity, selectivity, and antifouling properties [13]. 

TFN membranes with excellent performance have been exploited by utilizing various nanomaterials (such as metal–organic frameworks (MOFs), covalent organic frameworks (COFs), silica, titanium dioxide, carbon nanotubes (CNTs), graphene oxide (GO), g-C_3_N_4_ nanosheets, molybdenum disulfide, etc.) [14,15,16,17,18]. It is well known that a TFN membrane with high separation performance can be obtained through the optimization of the surface properties (hydrophilicity, charge, roughness, and interactions) or structure (including pore size, porosity, tortuosity, and thickness) of the thin film layer [19,20]. For example, CNTs are frequently applied to develop advanced TFN membranes because they can provide a one-dimensional fast water transport path [21]. After brush painting a single-walled carbon nanotube (SWCNT) network on membrane support, the resultant polyamide nanofiltration membranes can exhibit an extremely high water permeability of 40 L m^−2^ h^−1^ bar^−1^ and a high Na_2_SO_4_ rejection of 96.5% [22]. However, the CNTs are soft and have a high draw ratio, which may impair the water permeability of the membrane. In addition, the development of TFN membranes is currently limited by the poor dispersion of nanoparticles and the weak adhesion between nanoparticles and polymer matrix.

HNTs are natural aluminosilicate clay materials with tubular and hollow microstructures [23,24,25]. Unique among carbon nanotubes (CNTs), they are low-cost, available, and environmentally friendly materials for water treatment [26,27,28]. In addition, there are plenty of Al–OH groups on the internal lumen surface, which provides a considerable number of active sites for surface functionalization [12,29]. A previous study demonstrated that HNTs can be used to construct TFN membranes that act as nano-fillers or carriers and can endow these membranes with enhanced water permeability and selectivity [30,31,32]. Additionally, HNTs feature electropositive inner surfaces and electronegative outer surfaces, with great potential to repulse negative dyes such as Reactive Red during salt/dye separations through the Donnan effect. On the other hand, zeolitic imidazolate framework-8 (ZIF-8), which is composed of tetrahedral ions (Zn^2+^) and imidazole-derived organic linkers, is a commonly utilized water-stable MOF material that possesses a uniform pore size and high porosity available for the sharp molecular sieving of large molecules and fast water transport [33]. Hypothetically, taking advantage of the electronegative tube wall character of HNTs and the high porosity of ZIF-8, most negative dyes can be efficiently rejected, and water transfer can be significantly improved. Based on this assumption, a reasonable combination of HNTs and ZIF-8 may provide an alternative route to enhancing the comprehensive salt/dye separation performance of TFN membranes.

In this work, considering the poor dispersion of nanoparticles in TFN membranes, HNTs and ZIF-8 were first modified using poly (sodium p-styrenesulfonate) with a large number of hydrophilic groups to enhance their dispersion in water [34] so that the hydropathic properties of the membranes could be well controlled by adjusting the m-ZIF-8 and m-HNT contents. A series of modified ZIF-8/modified HNT/polyacrylonitrile (labeled as m-ZIF-8/m-HNT/PAN) TFN membranes were prepared, and the modulation of the separation performance of these TFN membrane sheets was achieved. The results demonstrated that HNTs can improve high water permeability by providing extra water pathways. Meanwhile, ZIF-8 can endow a membrane with enhanced separation because of its excellent molecular sieving ability. As verified by our experimental characterizations, the morphologies of the selective layers of TFN membranes can be tailored by changing the mass content of nanomaterials ranging from zero-dimensional m-ZIF-8 to one-dimensional m-HNTs. Therefore, the properties of these TFN membranes, including permeability and selectivity, can be also tuned and controlled.

## 2. Experimental Section

### 2.1. Materials and Chemicals

HNTs were provided by Henan Xiang Hu Environmental Protection Technology Co., Ltd., Zhengzhou, China. 2-Methylimidazole (Hmim), zinc nitrate hexahydrate (Zn(NO_3_)_2_·6H_2_O), and poly(sodium-p-styrenesulfonate) (PSS) (MW = 70 kDa) were purchased from J&K Scientific Ltd. (Shanghai, China). Disodium hydrogen phosphate sodium (Na_2_HPO_4_), triethylamine (TEA), dihydrogen phosphate sodium chloride (NaH_2_PO_4_), and glutaraldehyde (GA, 50% solution in water) were all acquired from Tianjin Kemiou Chemical Reagent Co., Ltd. (Tianjin, China). A polyacrylonitrile (PAN) ultrafiltration membrane (molecular weight cut off, MWCO = 50 kDa) was obtained from Sepro Mineral Systems Corp. (Langley, BC, Canada).

### 2.2. Synthesis of ZIF-8

ZIF-8 was prepared according to our previous report with a rapid room temperature synthesis method [35]. First, Zn^2+^ aqueous solution (4.93 mmol, 100 mL DI water) and Hmim aqueous solution (79.03 mmol, 100 mL DI water) were prepared. Then, TEA (79.06 mmol) was added to the Hmim aqueous solution to achieve the deprotonation of the Hmim. Subsequently, the deprotonated Hmim solution and the Zn^2+^ solution were mixed under vigorous stirring for 10 min. Following that, the same amount of TEA was added again at a molar ratio of 1:16:32 for Zn^2+^:Hmim:TEA in the final mixture. After an hour, the precipitate was collected and washed with DI water thrice. Finally, ZIF-8 nanomaterials were obtained after completely drying them in a vacuum oven at 60 °C.

### 2.3. Modification of ZIF-8 and HNTs with PSS

The modification of ZIF-8 or HNTs with PSS was performed by dispersing 0.2 g of nanomaterials (ZIF-8 or HNTs) in 50 mL of 2 wt% PSS aqueous solution with magnetic stirring for 24 h. The acquired suspension was left for 2 h, and then, the supernatant was collected via centrifugation at 3000 rpm for 10 min. The modified ZIF-8 (m-ZIF-8) or the modified HNTs (m-HNTs) could be collected via washing with DI water thrice followed by drying in a vacuum oven at 60 °C.

### 2.4. Preparation of m-ZIF-8/m-HNT/PAN Nanocomposite Membranes

First, 5 mg mL^−1^ of m-ZIF-8 and m-HNTs aqueous solution was prepared by dispersing the corresponding nanomaterials in DI water and sonicating for 10 min. Also, 0.2 wt% PVA aqueous solution was obtained by dissolving 0.2 g of PVA in 99.8 g of DI water at 90 °C for 4 h. To fabricate the m-ZIF-8/m-HNTs/PAN nanocomposite membranes, a certain volume of the m-ZIF-8 and m-HNTs aqueous solution and 1 mL of 0.2 wt% PVA aqueous solution were mixed and poured on the PAN membrane substrate, followed by evaporating the solvent at 80 °C. Finally, it was immersed in 0.2 wt% GA aqueous solution at 80 °C for about 30 min to achieve further cross-linking.

In order to analyze the effects of the used nanomaterials on membrane structures, chemical composition, separation performance, and antifouling performance, m-ZIF-8/m-HNT/PAN membranes with different mass amounts of m-ZIF and/or m-HNTs were fabricated and denominated as follows:

### 2.5. Characterization of HNTs, m-HNTs, ZIF-8, and m-ZIF-8

Nano-materials, including HNTs, m-HNTs, ZIF-8, and m-ZIF-8, were characterized with high-resolution transmission electron microscopy (HRTEM, JEM-2100, JEOL, Tokyo, Japan), Fourier-transform infrared spectroscopy (FTIR, Nicolet IS10, Thermo Scientific, Waltham, MA, USA), and X-ray powder diffraction (XRD, D8 Advance, Bruker, Mannheim, Germany). HRTEM images were acquired to examine the microstructures. FTIR was used to obtain the chemical composition information in a range of 4000~400 cm^−1^. In addition, the XRD pattern was recorded at room temperature with Cu Kα radiation at a scan rate of 10 °C min^−1^ in a range of 5°~80° to analyze its crystalline properties. 

### 2.6. Characterization of the m-ZIF-8/m-HNT/PAN Membranes

The m-ZIF-8/m-HNT/PAN membranes were characterized via field emission scanning electron microscopy (FESEM, JSM-7001F, JEOL, Japan), XRD (D8 Advance, Bruker, Germany), attenuated total reflection Fourier-transform infrared spectroscopy (ATR-FTIR, Nicolet IS10, Thermo Scientific, USA), and water contact angle measurements. Each membrane sample for SEM measurement was frozen via liquid nitrogen first to preserve the integrity of the membrane structure. Then, the SEM images were obtained at an accelerating voltage of 10 kV. ATR-FTIR spectra could be obtained by utilizing an attenuated total reflectance (ATR) unit to scan ten times in a range of 4000~400 cm^−1^. The XRD test conditions for the membranes were the same as for the nanomaterials. Water contact angle tests were employed to evaluate membrane hydrophilicity using a contact angle goniometer (OCA20, Dataphysics Instruments, Stuttgart, Germany) via the sessile drop method.

### 2.7. Filtration Tests of the m-ZIF-8/m-HNT/PAN Membranes

As the two key parameters of evaluating membrane properties, cross-flow filtration was conducted to evaluate the permeability and selectivity of those as-prepared TFN membranes. Each membrane was kept at 0.8 MPa for 30 min, and then, the pressure was changed to 0.4 MPa to evaluate the filtration performance. Water flux (*J*, L m^−2^ h^−1^ MPa ^−1^) for the fabricated membranes can be calculated according to Equation (1).

(1) $$J=\frac{V}{A\cdot \Delta t\cdot P}$$

where Δ*V* is the permeate volume (m^3^), *A* is the effective membrane area (12.57 cm^2^), Δ*t* is the filtration time (h), and Δ*P* is the trans-membrane pressure difference (MPa). 

Rejection (*R*, %) for several salts or dyes, such as NaCl, Na_2_SO_4_, MgSO_4_, MgCl_2_, and Reactive Red 49, can be achieved by utilizing 0.5 g L^−1^ of aqueous solution as the feed. Equation (2) is employed to calculate the corresponding rejection:

(2) $$R=\left(1-\frac{C_{P}}{C_{f}}\right)\times 100\%$$

where *C_p_* and *C_f_* are the concentrations of the feed (*C_f_*, mol L^−1^) and permeate (*C_p_*, mol L^−1^), respectively. The salt concentration was determined via the linear relationship between concentration and conductivity, while the dye concentration was determined via the linear relationship between concentration and absorbance at an optimal absorption wavelength of 523.5 nm.

## 3. Results and Discussion

### 3.1. Characterization of the m-HNTs and m-ZIF-8 Nanomaterials

**Figure 1:**
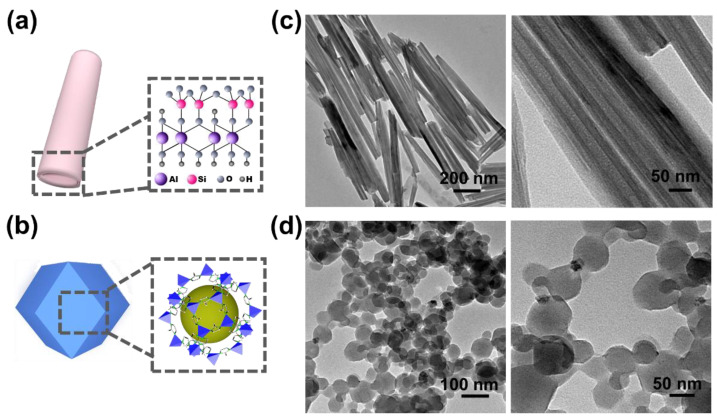
Structures of HNTs (**a**) and ZIF-8 (**b**) and HRTEM images of m-HNTs (**c**) and m-ZIF-8 (**d**).

Figure 1a,b schematically show the detailed structure of HNTs and ZIF-8, respectively. Generally, HNTs can be dispersed in an aqueous solution for several hours, while ZIF-8 will agglomerate because of a lack of hydrophilic groups. Herein, PSS, with plenty of hydrophilic groups, is utilized to enhance the dispersion of HNTs and ZIF-8 in water [36]. Figure 1c,d illustrate HRTEM images of m-HNTs and m-ZIF-8 at different magnifications. It was apparent that the modification of the PSS did not affect the original structure of the HNT or ZIF-8 materials.

**Figure 2:**
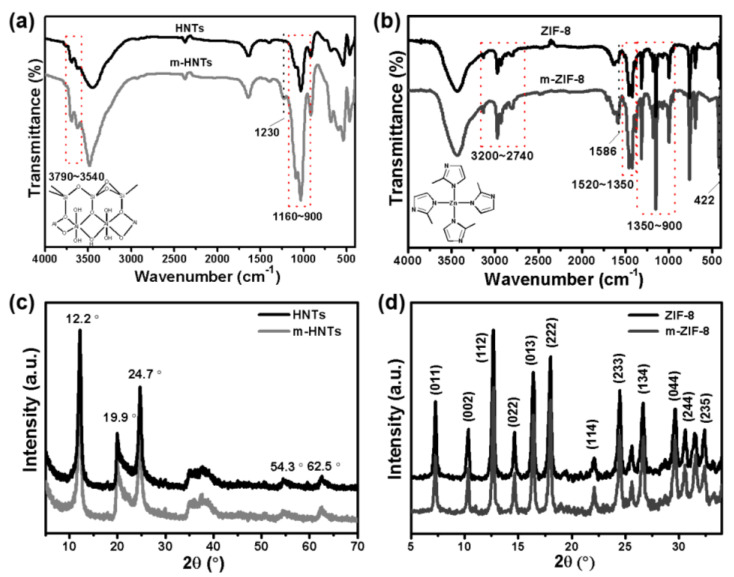
(**a**,**b**) FITR spectra and (**c**,**d**) XRD patterns of the HNTs and ZIF-8 before or after modification via PSS.

Figure 2a demonstrates the FTIR spectra of the HNTs and m-HNTs. The peaks between 3790~3540 cm^−1^ refer to the stretching vibrations of O-H bonds in the external (Si-O-H) or internal surface (Al-O-H), and those between 1160~900 cm^−1^ correspond to the stretching vibrations of O-Si-O or O-Al-O bonds [37]. The small peak at 1230 cm^−1^, which can be indexed to the stretching vibration of the –SO_3_Na groups, suggested the successful modification of PSS [38]. In other words, PSS can be adhered to the HNTs’ surfaces via electrostatic interactions. Figure 2b illustrates the FTIR spectra of ZIF-8 and m-ZIF-8. The peaks at 3200~2740 cm^−1^ can be ascribed to the stretching vibration of C-H in the Hmim [39]. Meanwhile, the stretching and in-plane or out-plane bending vibrations of the imidazole ring are located at around 1520~1350 cm^−1^ and 1350~900 cm^−1^, respectively [40]. A sharp peak at 422 cm^−1^ can be attributed to the stretching vibration of the Zn-N bond, indicative of the coordination of Hmin and Zn^2+^ [41]. Furthermore, compared with ZIF-8, m-ZIF-8 demonstrates much stronger peak intensities between 1350~900 cm^−1^ given the existence of –SO_3_Na groups. Figure 2c,d show the XRD patterns of HNTs, m-HNTs, ZIF-8, and m-ZIF-8. Both HNTs and m-HNTs demonstrate characteristic diffraction reflections at 12.2°, 19.9°, 24.7°, 54.3°, and 62.5° [42], and there are no obvious differences between these two XRD patterns. Similarly, the as-synthesized ZIF-8 and m-ZIF-8 also showcase characteristic peaks at 7.4°, 10.5°, 12.6°, 14.7°, 16.4°, 18.0°, 22.1°, 24.4°, 26.6°, 29.6°, 30.6°, and 32.4°, which correspond to the (011), (002), (112), (002), (013), (222), (114), (223), (134), (004), (244), and (235) crystal planes, respectively [43]. All the results demonstrate that the PSS was attached to the HNT or ZIF-8 surfaces via electrostatic interaction, and the modification of PSS did not affect the original crystalline structures of these two materials. 

### 3.2. Characterization of the m-ZIF-8/m-HNT/PAN Membranes

**Figure 3:**
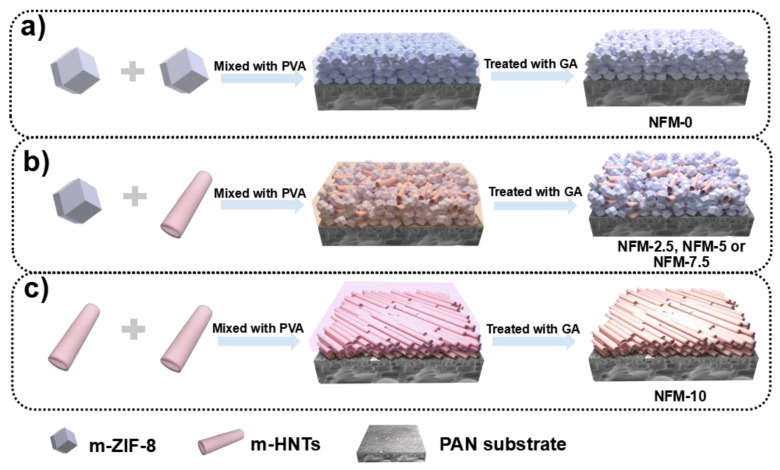
Schematic diagrams for preparing the NFM-X series (**a**) NFM-0, (**b**) NFM-2.5, NFM-5 or NFM-7.5, and (**c**) NFM-10.

The m-ZIF-8/m-HNT/PAN membranes could be fabricated by coating the mixtures of m-ZIF-8 and/or m-HNTs and PVA on the PAN substrate, followed by cross-linking with glutaraldehyde, as schematically shown in Figure 3. The obtained membrane is denoted as NFM-X (X = 0, 2.5, 5, 7.5, and 10, where X refers to the loading mass amount of m-HNTs), and the detailed composition of these membranes is illustrated in Table 1.

**Figure 4:**
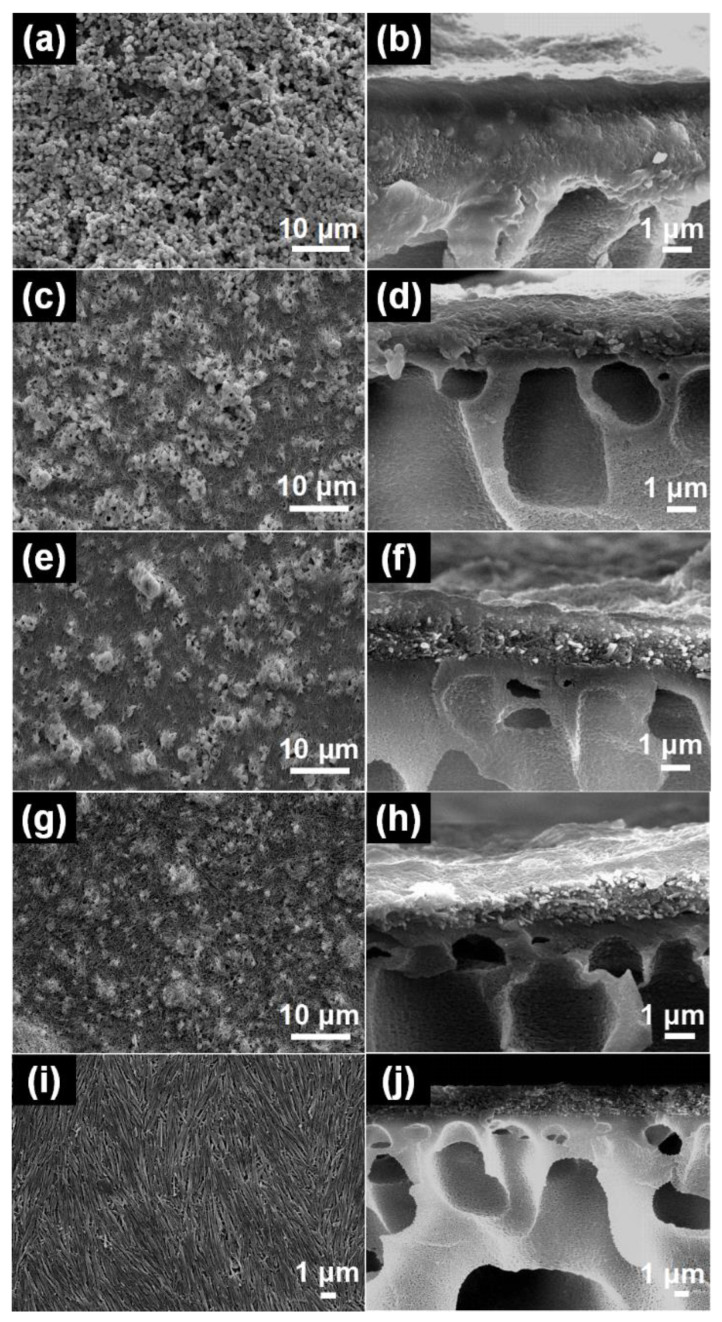
SEM images of membrane surfaces and cross-sections of NFM-0 (**a**,**b**), NFM-2.5 (**c**,**d**), NFM-5 (**e**,**f**), NFM-7.5 (**g**,**h**), and NFM-10 (**i**,**j**) membranes.

Figure 4 illustrates the surface and cross-section morphologies for the NFM-0, NFM-2.5, NFM-5, NFM-7.5, and NFM-10 membranes. As shown in Figure 4a,b, all the m-ZIF-8 nanoparticles can be uniformly spread out on the PAN substrate. However, some small protrusions that were constructed by m-ZIF-8 aggregates still exist. The formation of these aggregates may be attributed to the decrease in free surface energy caused by intermolecular interactions such as Van der Waals forces, electrostatic interactions, and so on [44,45]. These interactions jointly formed a short-range driving force that helped to accelerate the m-ZIF-8’s self-assembly into clusters during solvent evaporation. As shown in Figure 4c,e,g, when 2.5 mg, 5 mg, and 7.5 mg of m-HNTs were added to replace an equal amount of m-ZIF-8, the corresponding membranes (NFM-2.5, NFM-5, and NFM-7.5) demonstrated similar surface morphologies. In the meantime, as the number of m-HNTs increased, the size of the m-ZIF-8 aggregates gradually declined, suggesting that the introduction of m-HNTs can effectively inhibit m-ZIF-8 cluster growth, possibly because of the overall increased electrostatic repulsion energy, which determined a finite aggregation. From the cross-section image shown in Figure 4f, it can be clearly observed that m-ZIF-8 is more likely to gather near the air side, while the m-HNTs tend to gather on the other side. This can be explained by the factor of gravity. Compared with m-ZIF-8 (bulk density ~0.35 g cm^−3^, average size < 100 nm) [46], m-HNTs with a higher density (~2.53 g cm^−3^, a length of 500~600 nm) [47] are prone to sinking into the PAN substrate more quickly. Figure 4i,j show the morphology of the membrane that was prepared using pure m-HNTs without the addition of m-ZIF-8. The surface morphology of the membranes showed that the m-HNTs were uniformly dispersed on the PAN substrate. Unlike the NFM-0 membranes, no obvious protrusions of m-HNTs were observed on either the surfaces or cross-sections of the NFM-10 membranes.

**Figure 5:**
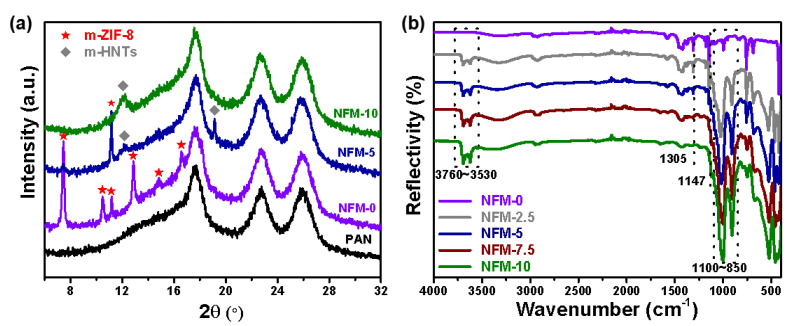
XRD pattern (**a**) and ATR-FTIR spectra (**b**) of the PAN and NFM-X membranes.

Furthermore, XRD measurement was implemented to analyze the crystalline properties of the NFM-0, NFM-5, and NFM-10 membranes. As presented in Figure 5a, more new diffraction reflections, attributable to the applied nanomaterials, appear in these membranes compared with the PAN substrate, demonstrating the successful introduction of the m-HNT and m-ZIF-8 nanomaterials. The ATR-FTIR spectra of the NFM-0, NFM-2.5, NFM-5, NFM-7.5, and NFM-10 were then measured and are illustrated in Figure 5b. Compared with the NFM-0 membrane, all the NFM-X membranes demonstrated two obvious peaks at 3697 cm^−1^ and 3623 cm^−1^, which can be attributed to the stretching vibrations of Al-OH or Si-OH groups on m-HNTs [48]. The peaks at around 1100~850 cm^−1^ can be ascribed to the stretching vibrations of O-Si-O and O-Al-Si groups on m-HNTs [49]. It can be seen that, with the gradual replacement of m-ZIF-8 by m-HNTs in the corresponding membranes, the intensities of the peaks at 3760~3530 cm^−1^ and 1100~850 cm^−1^ are also enhanced. Furthermore, the in-plane bending vibrations of the imidazole ring at 1305 cm^−1^ and 1147 cm^−1^ became weak. 

### 3.3. Hydrophilicity of the m-ZIF-8/m-HNT/PAN Membranes

**Figure 6:**
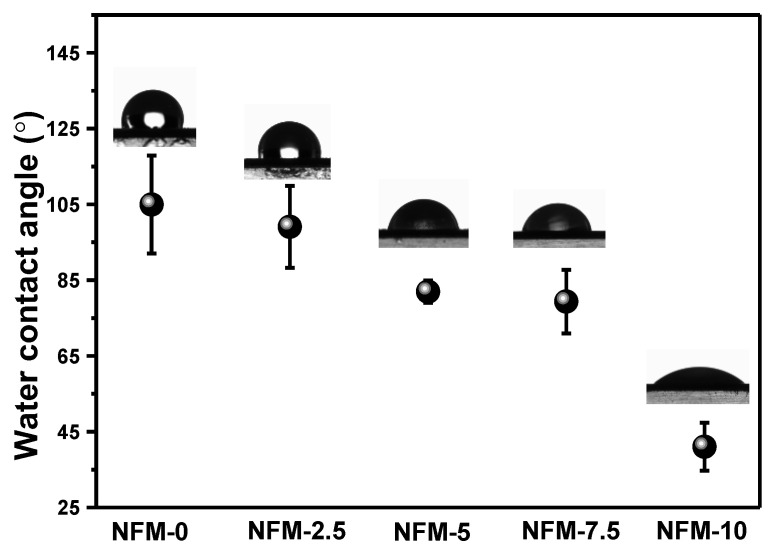
Water contact angle results for the NFM-0, NFM-2.5, NFM-5, NFM-7.5, and NFM-10 membranes.

The hydrophilicity of membranes is one of the critical parameters for indexing their permeability, selectivity, and antifouling performance. Herein, the hydrophilicity of the membranes is evaluated via the water contact angle, as shown in Figure 6. The NFM-0 membrane, which was fabricated by coating the m-ZIF-8 nanomaterials on the PAN substrate, illustrates an average water contact angle of 104.97°. Compared with NFM-0, the rest of the NFM-X membranes showcase lower water contact angles that can be placed in the following sequence: NFM-0 > NFM-2.5 > NFM-5 > NFM-7.5 > NFM-10. This suggests that the existence of m-HNTs can endow TFN membranes with better hydrophilicity. Intrinsically, the hydropathy properties of these TFN membranes can be controlled by altering the number of these two nanomaterials. As frequently reported [50], variations in the water contact angles of membranes are closely related to membrane surface roughness and functional groups, and a low surface roughness generally results in a high water contact angle. In our case, the NFM membrane surface gradually became smooth with the addition of HNTs, as demonstrated by Figure 4, which should theoretically enlarge the water contact angle and is the opposite of the trend observed. Therefore, it can be speculated that the HNTs primarily dominated the overall membrane hydrophilicity since a large number of hydroxyl groups were introduced.

### 3.4. Separation Performance of the m-ZIF-8/m-HNT/PAN Membranes

**Figure 7:**
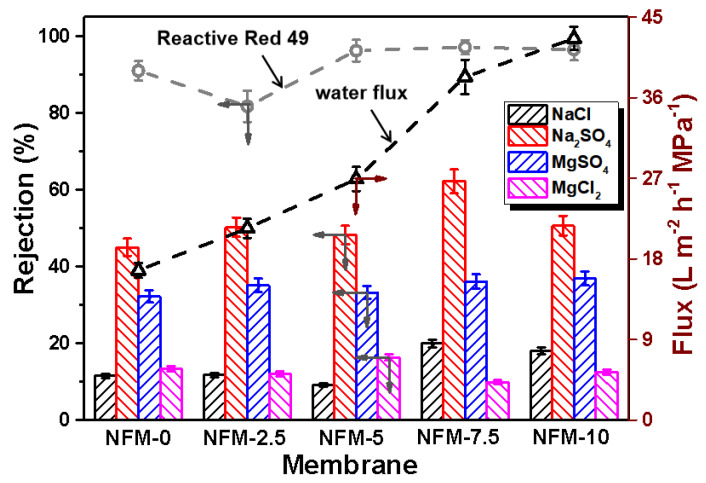
The pure water flux (black dotted line) and rejection of Reactive Red 49 (light gray dotted line) and salts for the NFM-0, NFM-2.5, NFM-5, NFM-7.5, and NFM-10 membranes.

**Figure 8:**
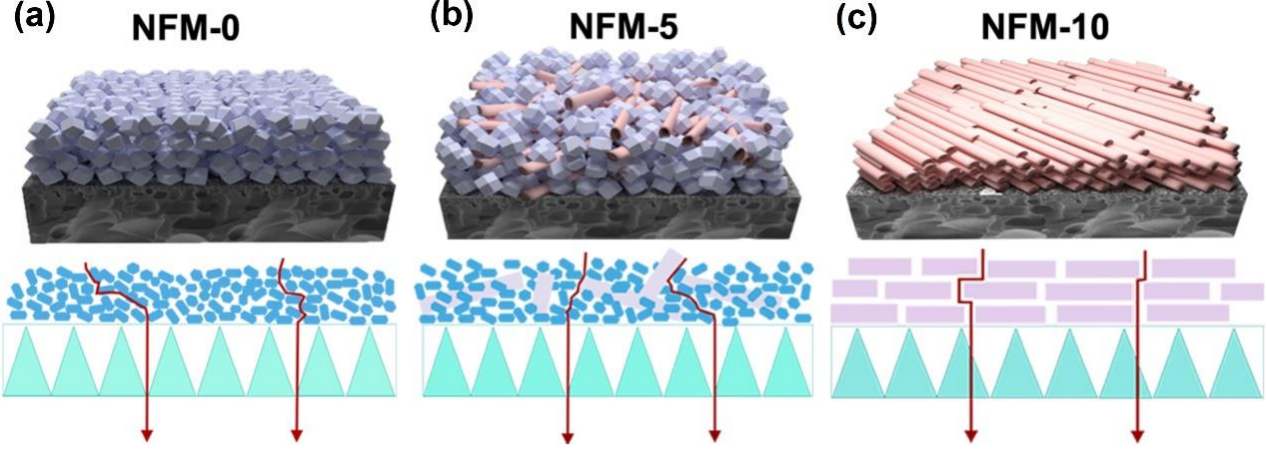
Schematic diagrams of the water transport paths for the NFM-0, NFM-5, and NFM-10 membranes.

Water flux and the rejection of salts and dyes were measured to estimate the separation performance of the m-ZIF-8/m-HNT/PAN membranes, as presented in Figure 7. Water fluxes for the NFM-0, NFM-2.5, NFM-5.0, NFM-7.5, and NFM-10 membranes were 16.7 L m^−2^ h^−1^ MPa^−1^, 21.4 L m^−2^ h^−1^ MPa^−1^, 26.9 L m^−2^ h^−1^ MPa^−1^, 38.3 L m^−2^ h^−1^ MPa^−1^, and 42.6 L m^−2^ h^−1^ MPa^−1^, respectively. Obviously, the water fluxes show the following sequence: NFM-0 < NFM-2.5 < NFM-5 < NFM-7.5 < NFM-10. This is clearly related to the hydrophilicity and structure of the TFN membranes. Specifically, the water flux of the series of TFN membranes was negatively related to the water contact angles. The layer structure of the TFN membranes was also vital. As can be seen in Figure 4 and Figure 8, the water path generated by small m-ZIF-8 nanoparticles was zigzag, long, and narrow for the NFM-0 membrane. Therefore, water molecules were subject to great resistance, hindering the water penetration through the NFM-0 membrane and resulting in low water flux. By contrast, for the NFM-10 membrane, the water path generated by aligned m-HNTs was regular, short, and interconnected. Thus, the water flux was higher than that of the NFM-0 membrane. The water flux of the NFM-2.5, NFM-5, and NFM-7.5 membranes, all composed of two nanomaterials, was intermediate. This scenario has also been reported for other kinds of membranes. For instance, porous graphene oxide can endow the corresponding composite membrane with short transport channels and help water molecules to pass by, and thus, its water flux is higher than a membrane prepared with graphene oxide [51].

The rejection rates of the m-ZIF-8/m-HNT/PAN membranes for Reactive Red 49, NaCl, Na_2_SO_4_, MgCl_2_, and MgSO_4_ were also measured. All these membranes showed a high rejection rate (exceeding 80%) for Reactive Red 49. In addition to the NFM-2.5 membrane, which showed a slight decrease in dye rejection, other membranes showed an increase in the rejection of Reactive Red 49 with the addition of m-HNTs, and the highest rejection rate achieved was 97.1%. The relatively high retention rate for reactive dyes indicates that the pore size range of our prepared TFN membranes was approximate to the size of the reactive dyes, and the introduction of m-HNTs and m-ZIF-8 particles could not degrade the separation performance of the reactive dyes. Interestingly, rejection rates for the salts conformed with the following order: Na_2_SO_4_ > MgSO_4_ > NaCl (≈MgCl_2_). This mainly depended on the interactions of the sieve effect and the Donnan effect in the thin film layer of the TFN membrane. Table 2 illustrates the stock radii and hydrated radii of several ions [52]. If the surface charge in the membrane is neutral, considering *r*(Na^+^) < *r*(Mg^2+^), the rejection rate for Na_2_SO_4_ is lower than for MgSO_4_ (Na_2_SO_4_ < MgSO_4_). The rejection demonstrates a reverse sequence (Na_2_SO_4_ > MgSO_4_). According to the Donnan effect, negatively charged membranes tend to adsorb high-valent cations and repel high-valent anions. Therefore, the TFN membrane can attract stronger positive charges and possess a negatively charged surface. In addition, since *r*(SO_4_^2−^) > *r*(Cl^−^) and the membrane possesses a negatively charged surface, the rejection rate for SO_4_^2−^ is higher than for Cl^−^, resulting in Na_2_SO_4_ > NaCl and MgSO_4_ > MgCl_2_. The Na_2_SO_4_ retention of the NFM-2.5, NFM-5, and NFM-7.5 membranes, all composed of these two nanomaterials, was significantly enhanced by up to 62%, and the retention of other salts varied slightly. Overall, the introduction of hydrophilic m-HNTs and m-ZIF-8 greatly improved the water flux of TFN membranes by up to 38.3 L m^−2^ h^−1^ MPa^−1^ while also maintaining good dye retention (~97.1%) and stable salt retention. Table 3 summarizes the performance of TFN nanofiltration membranes containing other nanoparticles reported in other literature. Obviously, our m-ZIF-8/m-HNT/PAN membranes have both high water flux and good dye/salt retention.

## 4. Conclusions

In summary, we proposed a new combination of m-ZIF-8 and m-HNTs, attempting to address the poor dispersion of nanoparticles in TFN membranes, and prepared a series of m-ZIF-8/m-HNT/PAN membranes by using the drop-coating method. It was demonstrated that the morphologies of the thin film layer of TFN membranes can be readily changed, varying from zero-dimensional m-ZIF-8 to one-dimensional m-HNTs. Consequently, the performance of these TFN membranes, including water flux and dye rejection, can be also adjusted and controlled. The experimental results showed that the modified m-ZIF-8/m-HNT/PAN membranes increased water flux by up to 42.6 L m^−2^ h^−1^ MPa^−1^. All these membranes showed a high inhibition of Reactive Red 49 (more than 80%) and maintained good salt retention. In particular, the optimized NFM-7.5 membrane showed an optimized separation performance with a 97.1% rejection of Reactive Red 49 and a 21.3% retention of NaCl. We emphasize that the properties of nanomaterials, including dimensionality, hydrophilicity, etc., should be taken into serious consideration when preparing advanced TFN membranes. This work is expected to promote the application of HNT and MOF nanomaterials in membrane separation technology and provide a new way to solve the dispersion of nanofillers in TFN membranes.

## Figures and Tables

**Table 1:** The fabrication of m-ZIF-8/m-HNT/PAN membranes.

Samples	Total Number of Nanomaterials (mg)	m-HNTs (mg)	m-ZIF-8 (mg)
NFM-0	10	0	10
NFM-2.5	10	2.5	7.5
NFM-5	10	5	5
NFM-7.5	10	7.5	2.5
NFM-10	10	10	0

**Table 2:** The stock radii and hydrated radii of several ions.

Ions	*r_s_* (Å)	*r_H_* (Å)
Na^+^	1.84	3.58
Mg^2+^	3.47	4.28
Cl^−^	1.21	3.32
SO_4_^2−^	2.3	3.79

**Table 3:** Comparison of dye/salt separation performance of m-ZIF-8/m-HNT/PAN membranes with data from the literature.

Membranes	Dye Molecule	Dye Rejection (%)	NaCl Rejection (%)	Water Flux (L m^−2^ h^−1^)	Ref.
bisAPAF-TMC/PES	Direct Red 23	98.7	19.5	17.0	[53]
c-CNT@GO	Methyl Blue	94.1	1.3	26.3	[54]
Fe(III)-phos-(PEI)/HPAN	Methyl Blue	99.9	7.5	8.5	[55]
SMA-PEI/PES	Congo Red	99.7	2.4	23	[56]
M-PIP	Reactive black 5	99.0	11.0	26.4	[57]
TA-Fe^3+^/PES	Congo red	99.0	5	27.2	[58]
Catechin-chitosan/HPAN	Acid fuchsin	98.7	12.5	14.4	[59]
GA/PEI-M	Methyl Blue	90.0	5.0	25.5	[60]
S-EMT/PA-2	Methyl blue	98.9	28.4	24.4	[61]
m-ZIF-8/m-HNTs/PAN	Reactive Red 49	97.1	21.3	17.0	This work

## Data Availability

Data is contained within the article.
